# Redescriptions and lectotype designations of Central American species of *Phaenonotum* Sharp (Coleoptera, Hydrophilidae) based on the type material from the David Sharp collection

**DOI:** 10.3897/zookeys.579.7748

**Published:** 2016-04-11

**Authors:** Albert Deler-Hernández, Martin Fikáček

**Affiliations:** 1 Department of Zoology, Faculty of Science, Charles University in Prague, Vinicná 7, CZ–128 44,Praha 2, Czech Republic; 2 Department of Entomology, National Museum, Cirkusova 1740, Praha 9 – Horni Pocernice, CZ–193 00, Czech Republic

**Keywords:** Coelostomatini, morphology, Neotropical region, Sphaeridiinae, taxonomy

## Abstract

In order to understand the identity of the Central American species of the genus *Phaenonotum* Sharp, 1882, the type specimens of the species described by Sharp (1882) deposited in the David Sharp collection in the Natural History Museum in London have been re-examined. The following species are redescribed: *Phaenonotum apicale* Sharp, 1882, *Phaenonotum collare* Sharp, 1882, *Phaenonotum dubium* Sharp, 1882 (confirmed as junior synonym of *Phaenonotum exstriatum* (Say, 1835)), *Phaenonotum laevicolle* Sharp, 1882, *Phaenonotum rotundulum* Sharp, 1882 and *Phaenonotum tarsale* Sharp, 1882. Lectotypes are designated for *Phaenonotum apicale*, *Phaenonotum collare*, *Phaenonotum rotundulum* and *Phaenonotum tarsale*. External diagnostic characters and morphology of male genitalia are illustrated. A table summarizing diagnostic characters allowing the identification of the species is provided.

## Abstract (es)

Para entender la identidad de las especies del género *Phaenonotum* Sharp, 1882 de América Central, se han reexaminado los especímenes tipo de las especies descritas por Sharp (1882) depositadas en la colección de David Sharp, del Museo de Historia Natural en Londres. Las especies redescritas son: *Phaenonotum apicale* Sharp, 1882, *Phaenonotum collare* Sharp, 1882, *Phaenonotum dubium* Sharp, 1882 (confirmado como sinónimo más reciente de *Phaenonotum exstriatum* (Say, 1835)), *Phaenonotum laevicolle* Sharp, 1882, *Phaenonotum rotundulum* Sharp, 1882 y *Phaenonotum tarsale* Sharp, 1882. Se designan lectotipos para las especies *Phaenonotum apicale*, *Phaenonotum collare*, *Phaenonotum rotundulum* y *Phaenonotum tarsale*. Se ilustran los caracteres diagnósticos y la morfología de los genitales. Una tabla resumen con los caracteres diagnósticos para facilitar la identificación de las especies es ofrecida.

## Introduction

The genus *Phaenonotum* Sharp, 1882 was described by the British specialist on water beetles, David Sharp, in his treatment of the Central American hydrophilid fauna in the famous *Biologia Centrali–Americana*. Based on material from Mexico, Nicaragua, Guatemala, Costa Rica and Panama available to him, Sharp (1882) recognized and described six species of that genus, and also recognized that the North American species *Cyclonotum exstriatum* (Say, 1835) is congeneric. A few other species originally described in other genera were later assigned to *Phaenonotum* by other authors (Knisch 1924; Orchymont 1937) and few additional species were described subsequently from Brazil (Orchymont 1937, 1943), Argentina (Bruch 1915), Venezuela (Archangelsky 1989), U.S.A (Smetana 1978) and Cuba (Deler-Hernández et al. 2013). In addition, the monotypic genus *Hydroglobus* Knisch, 1921 from Argentina was considered a part of *Phaenonotum* by Archangelsky (1991), but this was not followed by subsequent authors (see e.g. Clarkson et al. 2014 for diagnostic characters between *Hydroglobus* and *Phaenonotum*). At present, *Phaenonotum* seems to occur exclusively in the Neotropical and southern Nearctic Region from where 18 species are described (Hansen 1999; Deler-Hernández et al. 2013). The identity of the only non-American species, *Phaenonotum africanum* Régimbart, 1907 from the island of Bioko in Guinean Gulf, Africa, is unclear and the species needs to be re-examined.

Despite being frequently collected, *Phaenonotum* species were never properly revised, and only the fauna of North America and Argentina (partly) were treated in details by modern authors (Smetana 1978; Archangelsky 1991). Hence, no information on morphology of the species or identity of their types was published for the majority of species after their original descriptions, which makes the identification of newly collected material almost impossible. The only species for which types were reexamined and redescriptions and/or illustrations published are *Phaenonotum argentinense* Bruch, 1915, *Phaenonotum regimbarti* Bruch, 1915, and *Phaenonotum exstriatum* (Say, 1835) and its synonyms (Smetana 1978; Archangelsky 1991). In addition, the lectotype of *Phaenonotum laevicolle* Sharp, 1882 was designated by Smetana (1976), but without providing any information about the identity of that species. Of the recently described species, photos of the habitus and genitalia, and some details on morphology of *Phaenonotum minor* Smetana, 1978 were published by Deler-Hernández et al. (2013). The assignment of *Phaenonotum caribense* Archangelsky, 1989 to *Phaenonotum* was found questionable based on preliminary molecular data (A. Deler-Hernández & V. Sýkora, unpubl. data).

In the course of the review of *Phaenonotum* from the Greater Antilles, it was necessary to study the identities of the Central American species of the genus described by D. Sharp in order to confirm or exclude their occurrence in the Caribbean islands. The type series of all species described by Sharp and deposited in the Natural History Museum in London were therefore re-examined. To facilitate future studies, the results of these studies are summarized in the present paper, providing the redescriptions and illustrations of the species examined. In needed cases, the lectotypes have been designated in order to fix the identity of the species for future studies.

## Material and methods

Habitus photographs were taken using Canon EOS 550D digital camera with attached Canon MP-E65mm f/2.8 1–5× macro lens, and subsequently adapted in Adobe Photoshop CS5. Drawings of male genitalia are based on photographs taken using Canon EOS 1100D digital camera attached to Olympus BX41 compound microscope and subsequently combined in Helicon Focus software. Scanning electron micrographs of lectotypes were taken using Hitachi S-3700N environmental electron microscope at the Department of Paleontology, National Museum in Prague, using the uncoated specimens in low vacuum regime. Morphological terminology follows Smetana (1978), Archangelsky (1989, 1991) and Deler-Hernández et al. (2013).

Part of the specimens including the lectotypes were dissected, their genitalia were mounted in an alcohol soluble Euparal resin on a small piece of glass attached to the same pin as the specimen.

All lectotypes designated were labeled with the following red label: “Lectotype [or Paralectotype] / *Phaenonotum* / species-name with author and year of description / des. Deler-Hernández”.

Under each species listed as material examined label data are given verbatim between quotes (“ ”), each line of text is separated by a slash with spaces on both sides (/) and the information of each label is separated by double slashes with space on both sides (//). Other data are in square brackets ([]).

Examined specimens are deposited in the following collections:

BMNH
 The Natural History Museum, London, U.K. (M. Barclay) 


NMPC
National Museum, Prague, Czech Republic (M. Fikáček) 


## Taxonomy

### Phaenonotum apicale

Taxon classificationAnimaliaColeopteraHydrophilidae

Sharp, 1882

**Figure 1.**
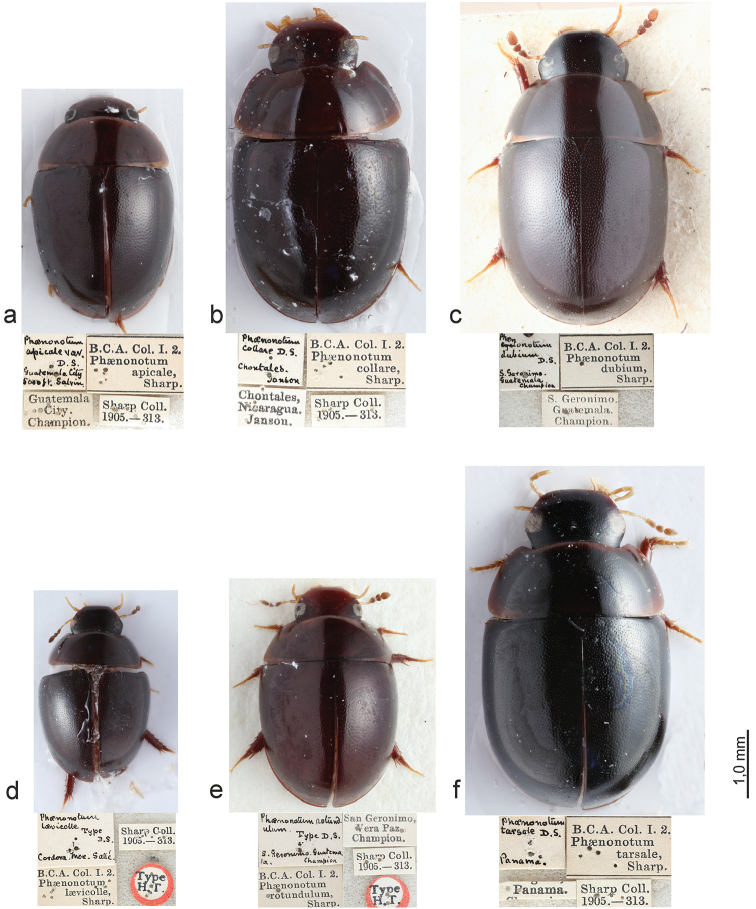
Habitus of type specimens (dorsal view) and original type labels. **a** *Phaenonotum apicale* Sharp (lectotype) **b** *Phaenonotum collare* Sharp (lectotype) **c** *Phaenonotum dubium* Sharp (lectotype) **d** *Phaenonotum laevicolle* Sharp (lectotype) **e** *Phaenonotum rotundulum* Sharp (lectotype) **f** *Phaenonotum tarsale* Sharp (lectotype).

**Figure 2.**
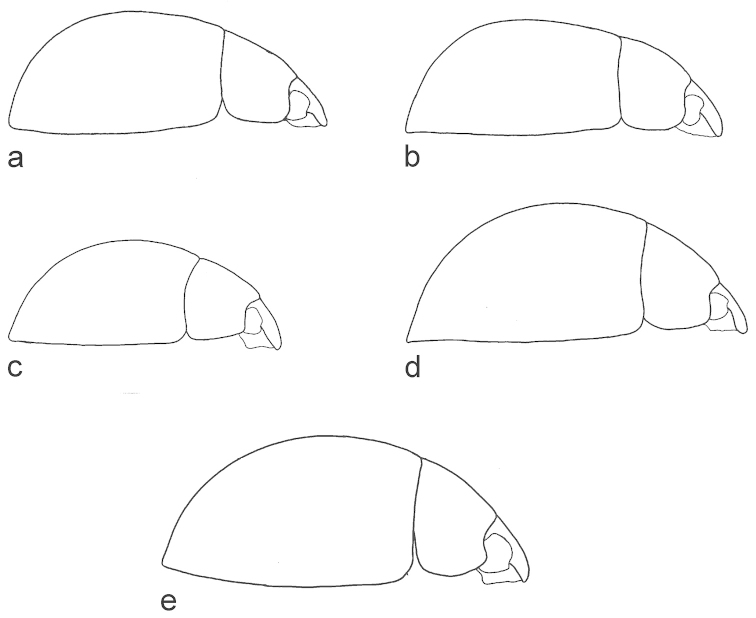
Habitus (lateral view). **a** *Phaenonotum apicale* Sharp (lectotype) **b** *Phaenonotum collare* Sharp (lectotype) **c** *Phaenonotum laevicolle* Sharp (lectotype) **d** *Phaenonotum rotundulum* Sharp (lectotype) **e** *Phaenonotum tarsale* Sharp (lectotype).

**Figure 3.**
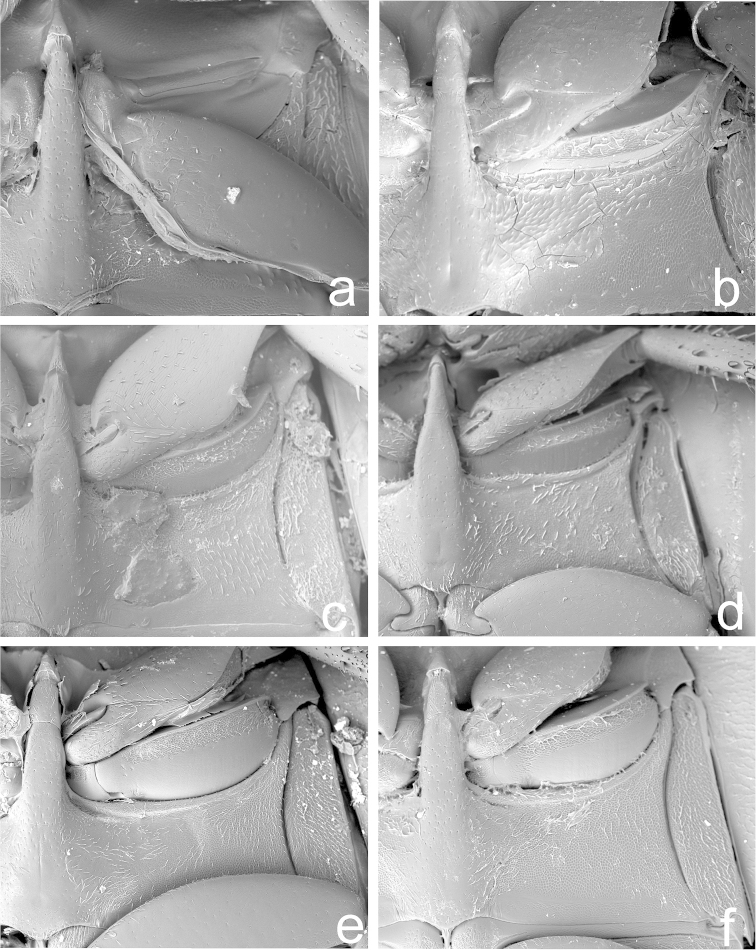
Meso-metaventral process. **a** *Phaenonotum apicale* Sharp (Lectotype) **b** *Phaenonotum collare* Sharp (Lectotype) **c** *Phaenonotum laevicolle* Sharp (Lectotype) **d** *Phaenonotum rotumdulum* Sharp (Lectotype) **e** *Phaenonotum tarsale* Sharp (Lectotype) **f** *Phaenonotum exstriatum* (Say).

**Figure 4.**
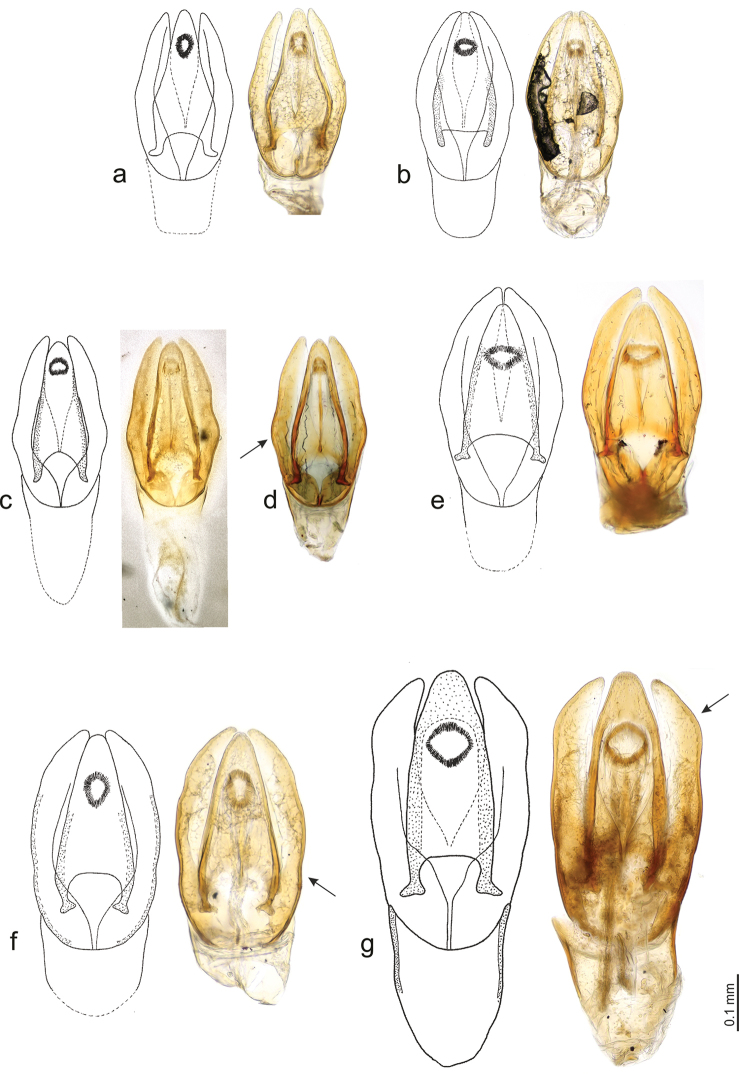
Aedeagus. **a** *Phaenonotum apicale* Sharp (Lectotype) **b** *Phaenonotum collare* Sharp (Lectotype) **c** *Phaenonotum dubium* Sharp (Lectotype) **d** *Phaenonotum exstriatum* (Say) (specimen from Haiti) **e** *Phaenonotum laevicolle* Sharp (Lectotype) **f** *Phaenonotum rotundulum* Sharp (Lectotype) **g** *Phaenonotum tarsale* Sharp (Lectotype).

Figures 1a , 2a , 3a , 4a

Phaenonotum apicale Sharp, 1882: 98.

#### Type material examined.

**Lectotype (hereby designated)**: male (BMNH): “Phaenonotum / apicale Var. / D.S. / Guatemala City. / 5000 ft. Salvin. // Guatemala City. Champion. // B.C.A. I. 2. / Phaenonotum / apicale, Sharp. // Sharp Coll. 1905.-313.” The specimen was re-mounted to a new label, with abdomen glued separately and aedeagus embedded in Euparal slide attached below the specimen. **Paralectotype**: female (BMNH): “Phaenonotum / apicale / Type / D.S. / Chontales, Nicaragua / Janson. // Chontales, / Nicaragua. / Janson. // B.C.A. Col. I. 2. / Phaenonotum / apicale, / Sharp. // Sharp Coll. / 1905.-313.”.

#### Other material examined.

1 unsexed specimen (BMNH): “Phaenonotum / apicale Var.? / David. Chiriqui / Champion // David, / Panama / Champion. // B.C.A. Col. I. 2. / Phaenonotum / apicale, / Sharp. // Sharp Coll. / 1905.-313.”; 1 unsexed specimen (BMNH): “Cuernavaca, / Morelos. / Hoge. // B.C.A. Col. I. 2. / Phaenonotum / apicale, / Sharp. // apicale / var, [hand written]”; 1 unsexed specimens (BMNH): “Tejupilco, Mex. / Temescaltepec / 18.vi.1933 [hand written] // H. E. Hilton, / R. L. Usinger / Collectors”; male (BMNH): “Tejupilco, Mex. / Temescaltepec / 18.vi.1933 [hand written] // H. E. Hilton, / R. L. Usinger / Collectors // Phaenonotum [hand written] / apicale Sharp [hand written] / J. Balfour-Brown det.”.

#### Type locality

(following lectotype designation). Guatemala City, 5000 feet [= 1525 m a.s.l.], Guatemala.

#### Redescription.

Habitus as in Figs 1a and 2a. Body length 2.9–3.2 mm (lectotype: 2.9 mm). Body form oval in dorsal view (Fig. 1a), elytra uniformly convex in lateral view (Fig. 2a). Dorsal surface dark brown (Fig. 1a). Antennae and maxillary palpi testaceous. Ventral surface reddish. Leg reddish, tarsomeres yellowish. Head and pronotum with fine and sparse punctures. Elytral punctation strongly impressed, coarser than pronotal and head punctation. Pronotum wider than long and convex. Epipleura very broad throughout. Meso- and metaventral processes fused into a common keel; mesoventral process arrow-head shaped with a distinct hood, as wide as metaventral process basally, metaventral process slender, parallel-sided, length of metaventrite medially (including metaventral process) ca. four times longer than mesoventral process; metathoracic discrimen indistinct (Fig. 3a). Profemora with long sparse pubescence in basal 0.75. Meso- and metafemora with very sparse and short pubescence only. All tarsi with long setae on ventral surface. Aedeagus (Fig. 4a) 0.4 mm long, with median lobe reaching apices of parameres; basal portion of median lobe angulate laterally, apical portion strongly narrowing; shape of the gonopore oval. Parameres wide and curved in median region. Phallobase not examined in detail.

#### Comments on lectotype designation.

Sharp (1882) mentions specimens from two localities: Nicaragua: Chontales and Guatemala: Guatemala City, but without specifying the number of specimens. In the Sharp collection, there are two specimens standing under the name of *Phaenonotum apicale*, one from each locality mentioned, and both corresponding with the data in the original description. We hence consider both as syntypes. The specimen from Guatemala City is the only male, and thus is designated here as lectotype, despite it appearing to be slightly teneral. Otherwise, there are four specimens from localities not corresponding to those given in the original description, which we do not consider as a part of the type series (see Other material examined).

### Phaenonotum collare

Taxon classificationAnimaliaColeopteraHydrophilidae

Sharp, 1882

Figures 1b , 2b , 3b , 4b

Phaenonotum collare Sharp, 1882: 99.

#### Type material examined.

**Lectotype (hereby designated)**: male (BMNH): “Phaenonotum / collare D.S. // Chontales, / Nicaragua. / Janson. // B.C.A. I. 2. / Phaenonotum / collare, / Sharp. // Sharp Coll. 1905.-313.”. The specimen was dissected, its abdomen is glued separately and the aedeagus is embedded in Euparal slide attached to the same pin. **Paralectotypes**: 1 female, 1 unsexed (BMNH): “Phaen / Cyclonotum / collare D.S. / Type / Chontales. Nicaragua. / Janson // B.C.A. I. 2. Phaenonotum / collare, Sharp. // Sharp Coll. 1905.-313.”; “Phaenonotum / collare D.S. / Chontales. Nicaragua. / Janson / B.C.A. I. 2. Phaenonotum / collare, Sharp. / Sharp Coll. 1905.-313.”.

#### Other material examined.

unsexed specimen (BMNH): “Phaenonotum / collare Var. / D.S. / El Zumbador / 2500 ft. Champion // El Tumbador, / Guatemala. / Champion. // B.C.A. I. 2. / Phaenonotum / collare, / Sharp. // Phaenonotum collare [hand written]”.

#### Type locality

(following lectotype designation). Chontales, Nicaragua.

#### Redescription.

Habitus as in Figs 1b and 2b. Body length 3.5–3.9 mm (lectotype: 3.9 mm). Body form oval in dorsal view (Fig. 1b), elytra less convex anteriorly and more convex posteriorly in lateral view (Fig. 2b). Dorsal surface brown (Fig. 1b). Antennae and maxillary palpi testaceous. Ventral surface reddish brown. Leg reddish, tarsomeres yellowish. Head with coarse and strongly impressed punctures. Pronotum with fine punctures, but sparser than head punctation. Elytral punctation (Fig. 1b) strongly impressed, punctures of the same size as on head and as coarse as head punctations. Epipleura very broad throughout. Meso- and metaventral processes slender and fused into a common keel; mesoventral process arrow-head shaped with an distinct hood, slightly wider than apex of metaventral process basally, metaventral process slender, nearly parallel-sided, only indistinctly narrowing anteriad, length of metaventrite medially (including metaventral process) ca. four time longer than mesoventral process; metathoracic discrimen distinct, forming a shallow impression basally (Fig. 3b). All tarsi with long setae on ventral surface. Aedeagus (Fig. 4b) 0.4 mm long, with median lobe reaching apices of parameres; basal portion of median lobe rounded laterally, apical portion widely rounded, median lobe narrowing towards apex; shape of the gonopore transversely oval. Parameres wide and slightly sinuate in median region. Phallobase as long as wide.

#### Comments on lectotype designation.

Sharp (1882) mentions specimens from two localities: Nicaragua: Chontales and Guatemala: El Tumbador, without specifying the numbers of specimens. Specimen(s) from Guatemala are assigned to the “var. *paulo angustior*” [= a little narrower], which excludes them from the type series based on Article 72.4.1 of the Code (ICZN 1999). In the Sharp collection, there are four specimens under the name *Phaenonotum collare*, three of which correspond to the Nicaraguan specimens mentioned in the original description, and one corresponding with “var. *paulo angustior*”. Only the specimens from Nicaragua are considered as part of the type series, and one of them, a dissected male, is designated as a lectotype, in order to fix the identity of the species for future studies.

### Phaenonotum dubium

Taxon classificationAnimaliaColeopteraHydrophilidae

 Sharp, 1882 (= Phaenonotum exstriatum (Say, 1835))

Figures 1c , 4c

Hydrophilus exstriatus Say 1835: 171. Trasferred to Phaenonotum by Sharp (1882: 98).
Phaenonotum dubium Sharp, 1882: 98. Synonymized with Phaenonotum exstriatum by Smetana (1978: 14).
Phaenonotum dubium For complete synonymy of Phaenonotum exstriatum see Hansen (1999).

#### Type material examined.

**Lectotype** (designated by Smetana 1978: 14): male (BMNH): “Phaen / cyclonotum / dubium / D.S. / S. Geronimo. / Guatemala / Champion // B.C.A. I. 2. / Phaenonotum / dubium, / Sharp. // S. Geronimo. / Guatemala / Champion. // LECTO- / TYPE [round label with purple margins]”. **Paralectotype**: 1 unsexed specimen (BMNH): “Phaen / Cyclonotum / dubium / Types / D.S. / S. Geronimo. / Guate- / mala. Champion. // B.C.A. I. 2. / Phaenonotum / dubium, / Sharp. // San Geronimo. / Vera Paz. / Champion. // Type / H. T. [round label with red margins]”.

#### Other type material.

Sharp (1882) also examined specimens from Mexico: Cordova, Vera Cruz, Oaxaca and Costa Rica: Cache, all of which have to be considered as paralectotypes. We did not examine these specimens.

#### Additional material examined.

male (dry-mounted) (NMPC): **CUBA**: **Granma Prov**: Cauto Cristo, Río Cauto, El Sitio, 01.v.2005, Coll. L. Chaves. male (dry-mounted) (BMNH): **HAITI**: Port au Prince, 1.iii.1908, Coll. Dr. M. Cameron, B. M. 1936-555. male (dry-mounted) (BMNH): **JAMAICA**: Kinstong, 16.ii.1908, Coll. Dr. M. Cameron. male (dry-mounted) (BMNH): **USA**: Delaware (ABTC000175) (NMPC).

#### Type locality.

San Geronimo, Guatemala.

#### Redescription.

Habitus as in Fig. 1c. Body length 3.5–3.7 mm (lectotype: 3.5 mm). Body form oval in dorsal view (Fig. 1c), elytra convex in lateral view. Dorsal surface dark brown (Fig. 1c). Antennae and maxillary palpi testaceous. Pronotum slightly paler than elytra. Ventral surface reddish brown. Leg reddish, tarsomeres yellowish. Head with fine and sparse punctures. Pronotum with punctures of same size as on head. Elytral punctation strongly impressed, much denser than on pronotum and head. Epipleura very broad throughout. Meso- and metaventral processes fused into a common keel; mesoventral process arrow-head shaped with an distinct hood, as wide as metaventral process basally, metaventral process slender, parallel-sided, length of metaventrite medially (including metaventral process) ca. four times longer than mesoventral process; metathoracic discrimen indistinct (Fig. 3f). Profemora with long sparse pubescence in basal 0.75. All tarsi with long setae on ventral surface. Aedeagus (Fig. 4c) 0.4 mm long, with median lobe reaching apices of parameres or nearly so; basal portion of median lobe nearly straight laterally, apical portion widely rounded, median lobe nearly of the same width throughout; shape of the gonopore transversely oval. Parameres strongly sinuate in median region. Phallobase as long as wide (Fig. 4d).

#### Comments on synonymy.

Examined type specimens of *Phaenonotum dubium* morphologically correspond with specimens of *Phaenonotum exstriatum* listed in “Additional material examined” in all characters, including morphology of the aedeagus and meso-metaventral process. Hence, we confirm that *Phaenonotum dubium* is a junior synonym of *Phaenonotum exstriatum*, as proposed by Smetana (1978).

### Phaenonotum laevicolle

Taxon classificationAnimaliaColeopteraHydrophilidae

Sharp, 1882

Figures 1d , 2c , 3c , 4e

Cyclonotum globulosum Mulsant, 1844: 167 (ascribed to Klug). [“Amérique méridionale”] (cf., Orchymont, 1937). Transferred to Phaenonotum by Knisch (1924: 114).
Phaenonotum laevicolle Sharp, 1882: 99. Considered as synonym of Phaenonotum globulosum by Orchymont (1937: 241). Synonymy not confirmed by subsequent authors.

#### Type material examined.

**Lectotype** (designated by Smetana 1976: 213): male (BMNH): “Phaenonotum / laevicolle / Type / D.S. / Cordova Mex Sallé. // B.C.A. I. 2. / Phaenonotum / laevicolle, / Sharp. // Sharp Coll. / 1905.-313.”. **Paralectotype: **male (BMNH): “Cubilguitz / Vera Paz. / Champion. // B.C.A. I. 2. / Phaenonotum / laevicolle, / Sharp”.

#### Other type material.

Sharp (1882) also examined specimens from Nicaragua: Chinandega, Managua and Chontales, all of which have to be considered as paralectotypes. We did not examine these specimens.

#### Type locality

(following lectotype designation). Cordova, Mexico.

#### Redescription.

Habitus as in Figs 1d and 2c. Body length 2.5–2.7 mm (lectotype: 2.7 mm). Body form oval in dorsal view (Fig. 1d), elytra evenly convex in lateral view (Fig. 2c). Dorsal surface brown (Fig. 1d). Antennae and maxillary palpi testaceous. Ventral surface reddish brown. Leg reddish, tarsomeres yellowish. Head with fine and sparse punctures. Pronotum with punctures of same size as on head. Elytral punctation strongly impressed, much coarser than pronotal and head punctation. Epipleura very broad throughout. Meso- and metaventral processes fused into a common keel; mesoventral process arrow-head shaped with narrow hood, its base narrower than apex of metaventrite; metaventral process stout, slightly widened subapically, length of metaventrite medially (including metaventral process) ca. three times longer than mesoventral process (Fig. 3c). All tarsi with long setae on ventral surface. Aedeagus (Fig. 4e) 0.5 mm long, with median lobe not reaching apices of parameres; basal portion of median lobe nearly straight laterally, apical portion widely rounded, median lobe narrowing towards apex; shape of the gonopore transversely subtriangular. Parameres wide and curved in median region. Phallobase not examined in detail.

#### Comments on synonymy.

Orchymont (1937) considered *Phaenonotum laevicolle* as a junior synonym of *Phaenonotum globulosum* described from Colombia, based on the study of the type specimens of both taxa. However, he only compared external characters used for diagnosis of *Phaenonotum* species at that time (i.e. dorsal punctation, length of tarsi), and did not study ventral morphology and male genitalia, which are crucial characters for species identification. Smetana (1976) reexamined the types of *Phaenonotum laevicolle* including genitalia, but he did not provide any comments on the synonymy proposed by Orchymont (1937), he neither studied the types of *Phaenonotum globulosum*. For that reason, the synonymy of *Phaenonotum laevicolle* with *Phaenonotum globulosum* needs to be confirmed by future studies.

### Phaenonotum rotundulum

Taxon classificationAnimaliaColeopteraHydrophilidae

Sharp, 1882

Figures 1e , 2d , 3d , 4f

Phaenonotum rotundulum Sharp, 1882: 100.

#### Type material examined.

**Lectotype (hereby designated)**: male (BMNH): “Phaenonotum rotund– / ulum. / Type D.S. / S. Geronimo. Guatema– / la. Champion // San Geronimo, / Vera Paz. / Champion. // B.C.A. I. 2. / Phaenonotum / rotundulum, / Sharp. // Sharp Coll. / 1905.-313. // Type / H.T. [round label with red margins].” We remounted the specimens, the abdomen is glued separately, and the aedeagus is embedded in a Euparal slide attached to the same pin. **Paralectotypes**: 1 unsexed specimen (BMNH): “Phaenonotum / rotundulum / D.S. / El Zumbador. / 2500 ft. Guate– / mala. Champion. // El Tumbador, / Guatemala. / Champion. // B.C.A. I. 2. / Phaenonotum / rotundulum, / Sharp. // Sharp Coll. / 1905.-313.”. 1 unsexed specimen (BMNH): “Phaenonotum / rotundulum / D.S. / Chacoj. Guatema / la. Champion // Chacoj, / R. Polochic, / Guatemala. / Champion // B.C.A. Col. I. 2. / Phaenonotum / rotundulum, / Sharp.”. 2 females (BMNH): same label data as the lectotype.

#### Other type material.

Sharp (1882) also examined specimens from Mexico: Cordova, Toxpam, Guatemala: San Juan, San Joaquin, Zapote, and Panama: Volcan de Chiriqui, 4000 to 6000 feet, all of which have to be considered as paralectotypes. We did not examine these specimens.

#### Type locality

(following lectotype designation). San Geronimo, Guatemala.

#### Redescription.

Habitus as in Figs 1e and 2d. Body length approximately 2.8–3.3 mm (lectotype: 3.3 mm). Body form oval in dorsal view (Fig. 1e), elytra highly and evenly convex in lateral view (Fig. 2d). Dorsal surface reddish brown (Fig. 1e). Antennae and maxillary palpi testaceous. Ventral surface reddish brown. Leg reddish, tarsomeres yellowish. Head with fine and sparse punctures. Pronotum with punctures of same size as on head. Elytral punctation strongly impressed, much coarser than on pronotum and head. Epipleura very broad throughout. Meso- and metaventral processes fused into a common keel; mesoventral process arrow-head shaped with indistinct hood, its base as wide as apex of metaventral process, metaventral process wide basally, strongly narrowing anteriad and hence triangular in shape, length of metaventrite medially (including metaventral process) ca. three time longer than mesoventral process; metathoracic discrimen weakly developed (Fig. 3d). Profemora with long sparse pubescence in basal 0.75. All tarsi with long setae on ventral surface. Aedeagus (Fig. 4f) 0.5 mm long, with median lobe not reaching apices of parameres; basal portion of median lobe curved laterally, apical portion widely rounded, median lobe narrowing towards apex; shape of the gonopore oval. Parameres slightly sinuate in median region. Phallobase not examined in detail.

#### Comments on lectotype designation.

Our request to borrow the Sharp specimens of *Phaenonotum rotundulum* resulted in the receipt of the above five specimens. These specimens, however, clearly represent only a smaller part of the type series, as many other localities were mentioned in the original description by Sharp (1882). All specimens examined agree with the data provided in the original description, and hence are clearly part of the type series. In order to fix the identity of the species for future studies, we are designating the dissected male labeled as “Type” as the lectotype of *Phaenonotum rotundulum*.

### Phaenonotum tarsale

Taxon classificationAnimaliaColeopteraHydrophilidae

Sharp, 1882

Figures 1f , 2e , 3e , 4g

Phaenonotum tarsale Sharp, 1882: 98.

#### Type material examined.

**Lectotype (hereby designated)**: male (BMNH): “Phaenonotum / tarsale D.S. / Panama. // B.C.A. Col. I. 2. / Phaenonotum / tarsale, / Sharp. // Panama. // Sharp Coll. / 1905.-313.” We remounted the specimen on a new label, with abdomen glued separately and aedeagus embedded in Euparal slide attached on the same pin. **Paralectotypes**: 1 male, 2 unsexed specimens (BMNH): same label data as the lectotype.

#### Type locality

(following lectotype designation). Panama.

#### Redescription.

Habitus as in Figs 1f and 2e. Body length 4.7–4.8 mm (lectotype: 4.8 mm). Body form oval in dorsal view (Fig. 1f), elytra highly and evenly convex in lateral view (Fig. 2e). Dorsal surface dark brown (Fig. 1f). Antennae and maxillary palpi testaceous. Pronotum slightly paler than elytra. Ventral surface reddish brown. Leg reddish, tarsomeres yellowish. Head with fine and sparse punctures. Pronotum with punctures of same size as on head, but slightly more sparsely than the head. Elytral punctation strongly impressed, much denser than on pronotum and head. Epipleura very broad throughout. Meso- and metaventral processes fused into a common keel; mesoventral process arrow-head shaped, very wide basally, slightly hooded apically, its base slightly wider than apex of metaventral process, metaventral process stout, parallel-sided, length of metaventrite medially (including metaventral process) ca. three time longer than mesoventral process; metathoracic discrimen weakly developed (Fig. 3e). Profemora with long sparse pubescence in basal 0.75. All tarsi with long setae on ventral surface. Aedeagus (Fig. 4g) 0.7 mm long, with median lobe slightly overlapping apices of parameres; basal portion of median lobe nearly straight laterally, apical portion widely rounded, median lobe nearly of the same width throughout; shape of the gonopore rounded. Parameres slightly sinuate in median region. Phallobase slightly longer than wide.

#### Comments on lectotype designation.

Our request to borrow the Sharp specimens of *Phaenonotum tarsale* resulted in the receipt of the above four specimens, all of them corresponding with the original description and clearly part of the type series. In order to fix the identity of the species for future studies, we are designating the dissected male as the lectotype of *Phaenonotum tarsale*.

## Discussion

The identification of species of *Phaenonotum* is a difficult task, due to the similarity of the species and the complicated process of finding relevant morphological characters. This may explain the absence of keys to *Phaenonotum* species. Studies on *Phaenonotum* from Central America, together with preliminary studies on this genus in the Caribbean and South America (Deler-Hernández, unpublished data) show that reliable identification is possible based on several external morphological characters, especially the morphology of the meso-metaventral process. This structure exhibits some variation between species, especially in the shape of the metaventral process, the width of the mesoventral process, and the “size” of the apical hood of the mesoventral process (Table 1; Fig. 3; figs 10–12 in Deler-Hernández et al. 2013; figs 230–231 in Smetana 1978). Male genitalia, though very similar at first view, provide the most important characters for species identification, such as the shape of the apex and the base of the median lobe, the shape and position of the gonopore, and the shape of the external margin of the parameres (Fig. 4). Body size is also helpful in some cases, allowing the separation of species with rather similar male genitalia. Traditional characters used by previous authors (e.g. Sharp 1882; Smetana 1978), i.e. the dorsal coloration and punctation of pronotum and elytra, are insufficient for a reliable identification, although may be helpful when used in combination with those of the meso-metaventral elevation and the aedeagus.

**Table 1.** Diagnostic characters of the *Phaenonotum* species described by D. Sharp.

	*Phaenonotum apicale*	*Phaenonotum collare*	*Phaenonotum exstriatum* (= *Phaenonotum dubium*)	*Phaenonotum laevicolle*	*Phaenonotum rotundulum*	*Phaenonotum tarsale*
Total body length	2.9–3.2 mm	3.5–3.9 mm	3.5–3.7 mm	2.5–2.7 mm	2.8–3.3 mm	4.7–4.8 mm
Shape of mesoventral process	arrow-head shaped with a distinct wide hood	arrow-head shaped with a distinct wide hood	arrow-head shaped with a distinct wide hood	arrow-head shaped with distinct narrow hood	arrow-head shaped with indistinct hood	arrow-head shaped, slightly hooded apically
Base of mesoventral process	as wide as apex of metaventral process	slightly wider than apex of metaventral process	as wide as apex of metaventral process	narrower than apex of metaventral process	as wide as apex of the metaventral process	slightly wider than apex of metaventral process
Metaventral process	slender, subparallel-sided	slender, subparallel-sided	slender, subparallel-sided	stout, slightly widened subapically	stout, wide basally, narrowing apically	stout, parallel-sided
Aedeagus: length of parameres	0.4 mm	0.4 mm	0.4 mm	0.5 mm	0.5 mm	0.7 mm
Aedeagus: length of median lobe	reaching apices of parameres	reaching apices of parameres	reaching apices of parameres or nearly so	not reaching apices of parameres	not reaching apices of parameres	slightly overlapping apices of parameres
Aedeagus: basal region of the median lobe laterally	angulate	rounded	nearly straight	nearly straight	slightly curved basally	nearly straight
Aedeagus: apical region of the median lobe	strongly narrowing	widely rounded	widely rounded	widely rounded	widely rounded	widely rounded
Aedeagus: shape of the parameres	wide and curved in median region	wide and slightly sinuate in median region	strongly sinuate in median region	wide and curved in median region	slightly sinuate	slightly sinuate
Aedeagus: shape of the gonopore	oval	transversely oval	transversely oval	transversely subtriangular	oval	rounded

## Supplementary Material

XML Treatment for Phaenonotum apicale

XML Treatment for Phaenonotum collare

XML Treatment for Phaenonotum dubium

XML Treatment for Phaenonotum laevicolle

XML Treatment for Phaenonotum rotundulum

XML Treatment for Phaenonotum tarsale
